# Carbon-Based Materials as Additives for Water-Based Drilling Fluids: Comparative Effects on Rheology, Filtration, and Shale Inhibition

**DOI:** 10.3390/ma19204288

**Published:** 2026-10-11

**Authors:** Mihail Tudose, Robert Costin Titei, Viorel Dragomir, Avram Lazăr, Ioana Gabriela Stan, Iuliana Veronica Ghețiu, Doru Bogdan Stoica, Alina Petronela Prundurel, Gheorghe Brănoiu, Sonia Mihai, Rami Doukeh

**Affiliations:** 1 Well Drilling, Extraction and Transport of Hydrocarbons Department, Petroleum-Gas University of Ploiesti, 39 Bucharest Blvd., 100680 Ploiesti, Romania; mihai_tud@yahoo.com (M.T.); titeirobert@yahoo.com (R.C.T.); viorel.dragomir@upg-ploiesti.ro (V.D.); avram_lazar55@yahoo.com (A.L.); dstoica@upg-ploiesti.ro (D.B.S.); alina.prundurel@upg-ploiesti.ro (A.P.P.); 2 Petroleum Geology and Reservoir Engineering Department, Petroleum-Gas University of Ploiesti, 39 Bucharest Blvd., 100680 Ploiesti, Romania; gheorghe.branoiu@upg-ploiesti.ro; 3 Petroleum Refining Engineering and Environmental Protection Department, Petroleum-Gas University of Ploiesti, 39 Bucharest Blvd., 100680 Ploiesti, Romania; smihai@upg-ploiesti.ro

**Keywords:** carbon-based materials, water-based drilling fluids, shale inhibition, linear shale swelling

## Abstract

Direct comparisons of structurally distinct carbon-based additives in water-based drilling fluids under identical conditions remain limited. This study evaluated grapevine-wood-derived carbon (GWC), graphitic carbon nitride (GCN), and commercial activated carbon (CAC), each incorporated at 20 g per formulation. Their structural, morphological, and textural properties were characterized, and their effects on rheology, API filtration, and 72 h linear shale swelling were assessed. GWC exhibited the highest DFT-derived specific surface area (939.17 m^2^ g^−1^) and, among the tested formulations, provided the best filtration performance, reducing fluid loss from 8.0 to 5.6 mL and mud-cake thickness from 1.5 to 0.5 mm. Although GCN possessed the lowest specific surface area (32.50 m^2^ g^−1^) and produced comparatively unfavorable filtration behavior, it produced the greatest reduction in shale swelling, decreasing final swelling from 12.2% to 7.3 ± 0.2%, corresponding to a 39.9% reduction relative to the carbon-additive-free reference formulation. GWC and CAC both limited swelling to 9.2%, while CAC produced intermediate filtration performance. These contrasting responses demonstrate that specific surface area and porosity alone do not determine additive performance. Among the tested formulations, GWC provided the best filtration control, whereas GCN produced the greatest reduction in shale swelling.

## 1. Introduction

Drilling fluids are essential to the safe and efficient construction of oil and gas wells, performing critical functions such as formation-pressure control, cuttings transport, drill-bit cooling, and wellbore stabilization [1]. Among the principal drilling-fluid systems, water-based drilling fluids are widely employed because of their operational versatility, comparatively low cost, and more favorable environmental profile than oil-based systems [2,3]. Nevertheless, maintaining suitable rheological behavior, limiting fluid invasion through the formation of a low-permeability filter cake, and controlling the hydration of reactive shale formations remain important formulation challenges [4,5,6]. The importance of limiting fluid invasion is further underscored by studies on fluid transport in porous and fractured rock. Under mechanical loading, the pore–fracture network evolves markedly; in coal subjected to uniaxial compression, fracture porosity increased from 0.44% to 5.01% at failure, and fractal analysis showed that permeability scales with the fourth power of the maximum pore diameter [7]. Temperature has also been identified as a key factor controlling seepage in fractured rock masses, shifting equilibrium states and facilitating abrupt changes in seepage behavior, with direct implications for petroleum exploitation [8]. Because near-wellbore stresses and downhole temperatures can thus enlarge flow pathways, effective filter-cake formation and pore plugging remain essential for wellbore stability. Consequently, increasing attention has been directed toward multifunctional additives capable of balancing these interrelated requirements while maintaining acceptable rheological behavior and overall drilling-fluid stability [9].

Within this broader search for multifunctional additives, carbon-based materials have emerged as a particularly promising class for water-based drilling fluids because of their potential to regulate rheology, improve thermal stability, reduce fluid loss, and enhance wellbore stability. In this context, previous studies have primarily examined engineered carbon nanomaterials, including carbon nanotubes, graphite nanoparticles, graphene, graphene oxide, and graphene nanoplatelets, whose performance has been associated with surface chemistry, dispersion state, particle–polymer interactions, pore plugging, and the formation of low-permeability filtration barriers [1].

Early studies particularly emphasized filtration control. Kosynkin et al. showed that a mixture of large-flake and powdered graphene oxide reduced API fluid loss and produced a filter cake approximately one order of magnitude thinner than that formed by a conventional bentonite–PolyPAC formulation [10]. Kusrini et al. similarly reported that graphene oxide decreased fluid loss from 6.0 to 3.6 mL after thermal conditioning at 135 °C. In contrast, phosphorylated graphene oxide caused a pronounced decrease in pH and substantially increased fluid loss, demonstrating that the effectiveness of carbon-based additives depends on their surface chemistry and compatibility with the drilling-fluid formulation [11]. Carbon materials may also modify interactions among suspended solids. Carboxylated carbon nanotubes were reported to limit bentonite-particle coalescence, improve solids-carrying capacity, and reduce fluid loss by 30.2% after aging at 180 °C [12]. Likewise, a hybrid system containing graphene nanoplatelets, silica nanoparticles, and graphite reduced fluid loss under both LTLP and HTHP conditions and decreased the dispersion of Woodford-shale cuttings through the formation of a compact particulate plugging network [13].

Biomass-derived carbon materials have subsequently been investigated. Hydrothermal carbon nanospheres prepared from glucose reduced filtration loss by 78% after aging at 220 °C and enhanced physical sealing when combined with polymeric additives [14]. Graphene nanoplatelets derived from waste coconut shells also improved filtration and reduced the linear swelling of Endau–Rompin shale, indicating the potential of biomass-derived carbon materials as multifunctional WBM additives [15]. Rana et al. further demonstrated the combined filtration and inhibition effects of SWCNTs/PVP nanocomposites, which reduced fluid loss by approximately 23%, increased shale recovery to 89.5%, and maintained recovery above 80% for up to 72 h. These improvements were attributed to pore blockage and the formation of a protective layer that restricted water access to the shale surface [16].

Most closely related to the present investigation, Ahmed et al. evaluated melamine-derived graphitic carbon nitride (g-C_3_N_4_) in a WBM. At a dosage of 2 g per 350 mL formulation, g-C_3_N_4_ decreased API fluid loss after hot rolling from 12.5 to 4.0 mL, reduced mud-cake thickness from 2.3 to 1.0 mm, and increased shale recovery from 88.0% to 99.6%. The accompanying decrease in the accessible surface area and pore volume of the treated shale was consistent with surface coverage and partial pore blocking by g-C_3_N_4_ [17].

Collectively, these findings demonstrate that carbon-based materials can influence rheology, filtration, and shale–water interactions, with the nature and magnitude of these effects depending on their composition, morphology, surface properties, and dispersion behavior. However, previous studies predominantly evaluated individual engineered nanomaterials or hybrid systems under different formulations and operating conditions. Consequently, comparative evidence concerning porous biomass-derived carbon, commercial activated carbon, and nitrogen-rich layered carbon nitride under identical experimental conditions remains limited. Accordingly, the present study compares grapevine-wood-derived carbon (GWC), graphitic carbon nitride (GCN), and commercial activated carbon (CAC), each added at 20 g per formulation, within the same WBM formulation and using identical preparation and testing conditions. This approach provides a consistent basis for relating their structural and textural characteristics to their effects on rheology, filtration, aqueous ionic composition, and long-term shale swelling.

## 2. Materials and Methods

### 2.1. Materials

Grapevine wood residues used as the carbonaceous precursor were collected from vineyards located in Ploiești, Romania. Melamine (99%) was purchased from Alfa Aesar (Ward Hill, MA, USA). Commercial activated carbon (product code CA0346) was supplied by Scharlau (Barcelona, Spain). High-purity nitrogen (Nitrogen 6.0, ≥99.999%) was supplied by Linde Gaz Romania (Timișoara, Romania). Absolute ethanol (99.5%) was obtained from Chimreactiv S.R.L. (Bucharest, Romania).

The commercial additives used in the preparation of the water-based drilling fluids (WBDFs), including a defoamer and a bactericide, were supplied by Newpark Romania (Ploiești, Romania). Barite (BaSO_4_), used as a weighting material, was obtained from RUA Bulgaria (Sofia, Bulgaria). Unless otherwise specified, all chemicals were of analytical grade and were used as received without further purification.

### 2.2. Preparation of the Carbon-Based Materials

#### 2.2.1. Preparation of Grapevine-Wood-Derived Carbon

The collected grapevine wood residues were dried to remove residual moisture and subsequently subjected to pyrolysis in a quartz-tube furnace under an inert nitrogen atmosphere. The furnace temperature was increased to 850 °C at a heating rate of 2 °C min^−1^ and maintained at this temperature for 10 h under a continuous nitrogen flow of 10 mL·min^−1^.

After completion of the pyrolysis process, the furnace was allowed to cool naturally to room temperature under continuous nitrogen flow to prevent oxidation of the carbonaceous product. The resulting grapevine-wood-derived carbon (GWC) was thoroughly washed with distilled water, followed by absolute ethanol, to remove water-soluble residues and loosely bound surface impurities. The recovered material was then dried at 100 °C for 3 h and stored in a sealed container until further characterization and use in the water-based drilling-fluid formulations.

#### 2.2.2. Synthesis of Graphitic Carbon Nitride (g-C_3_N_4_)

Graphitic carbon nitride (g-C_3_N_4_), hereafter denoted as GCN, was synthesized through the thermal polycondensation of melamine in a quartz-tube furnace under an inert nitrogen atmosphere. The furnace temperature was increased to 600 °C at a heating rate of 2 °C min^−1^ and maintained at this temperature for 6 h under a continuous nitrogen flow of 10 mL·min^−1^. This thermal treatment promoted the progressive condensation and polymerization of melamine, leading to the formation of graphitic carbon nitride [18,19].

After completion of the thermal treatment, the furnace was allowed to cool naturally to room temperature under continuous nitrogen flow. The resulting light-yellow g-C_3_N_4_ material was thoroughly washed with distilled water, followed by absolute ethanol, to remove residual soluble species and unreacted precursor. The washed material was subsequently dried at 100 °C for 3 h and stored in a sealed container until further characterization and use.

#### 2.2.3. Commercial Activated Carbon

Commercial activated carbon, hereafter denoted as CAC, was employed as a reference material for comparison with the grapevine-wood-derived carbon and synthesized g-C_3_N_4_. The commercial activated carbon was used as received without any additional chemical or thermal treatment.

### 2.3. Characterization of Carbon-Based Materials

The structural, morphological and textural characteristics of the investigated carbon-based materials were examined using complementary analytical techniques, including X-ray diffraction (XRD), scanning electron microscopy coupled with energy-dispersive X-ray spectroscopy (SEM–EDS), and nitrogen adsorption–desorption measurements. To ensure direct and reliable comparability with the characterization results reported in our previous studies [20,21], all measurements were performed using the same analytical instruments, experimental protocols, and operating conditions. This consistent characterization approach minimized methodological variability and enabled the observed differences among the investigated materials to be attributed primarily to their intrinsic structural, morphological, and textural properties.

The crystalline structure and phase composition of the materials were examined using a D8 Advance diffractometer (Bruker AXS, Karlsruhe, Germany) operated with Cu Kα radiation (λ = 1.5406 Å). Diffraction patterns were recorded in θ–θ geometry using a Bragg–Brentano optical configuration over a 2θ range of 10–60°. The measurements were conducted at a tube voltage of 40 kV and an anode current of 40 mA, using a step size of 0.1° (2θ) and a counting time of 5 s per step. Data acquisition was performed using XRD Commander v2.6.1. Crystalline phases were identified using Diffracplus EVA v14 in conjunction with the PDF-ICDD reference database, while quantitative phase analysis, where applicable, was carried out using Diffracplus TOPAS v4.1.

Surface morphology and elemental composition were investigated by SEM–EDS using a scanning electron microscope manufactured by FEI Company (Hillsboro, OR, USA). SEM imaging was used to examine the surface architecture, particle morphology, and dry-state aggregation characteristics of the samples, whereas EDS analysis provided information regarding their principal elemental constituents. For each material, elemental mapping and semi-quantitative EDS analysis were performed over one representative area, and the results are reported as atomic percentages (at.%).

The textural properties were determined from nitrogen adsorption–desorption measurements conducted at 77.35 K using a Quantachrome NOVA 2200e Gas Sorption Analyzer (Boynton Beach, FL, USA). The isotherms were recorded over a relative-pressure range of $p/p_{0}=0.005–1.0$, and the resulting data were processed using NovaWin v11.03. Before analysis, all samples were degassed under vacuum at 200 °C for 12 h. The specific surface area, total pore volume, and pore-size distribution were calculated using the quenched solid density functional theory (QSDFT) equilibrium kernel for N_2_ at 77 K on carbon, assuming slit-shaped pores, applied to the desorption branch of the isotherm (NovaWin v11.03). Because this kernel was developed for carbon surfaces, its application to GCN, a carbon nitride whose mesoporosity arises mainly from interparticle voids, should be regarded as an approximation. Each sample was measured once, and replicate measurements were not performed.

Collectively, these complementary analyses provided information on the crystalline structure, phase composition, surface morphology, elemental composition, specific surface area, pore volume, and pore-size distribution of grapevine-wood-derived carbon (GWC), graphitic carbon nitride (GCN), and commercial activated carbon (CAC).

### 2.4. Preparation of the Reference and Carbon-Based-Material-Modified Water-Based Drilling Fluids

Four water-based drilling fluids (WBM 0–WBM 3) were prepared using the compositions presented in Table 1. To maintain methodological consistency with our previous studies, all formulations were prepared strictly following the same experimental procedure, additive-incorporation sequence, mixing times, mixing speeds, and operational conditions described in our previously published work [20,21]. WBM 0, prepared without any carbon-based additive, was used as the reference formulation. WBM 1–WBM 3 were modified with 20 g of grapevine-wood-derived carbon (GWC), graphitic carbon nitride (GCN), or commercial activated carbon (CAC), respectively. Therefore, the principal compositional difference among the four formulations was the absence or type of carbon-based additive. The carbon-based materials were added to an otherwise identical base formulation; therefore, the absolute amounts of all other components were identical in WBM 0–WBM 3, whereas their mass fractions were approximately 1.6% (relative) lower in the carbon-modified fluids (Table 1). An inert particulate control at the same mass loading was not included; consequently, carbon-specific effects cannot be fully separated from the effect of the additional solids loading.

The dosage of 20 g per formulation (approximately 1.616 wt.%) was selected to maintain consistency with our previous investigations [20,21] and to evaluate the three additives at a common mass loading within the same base-fluid formulation. Although XRD, SEM–EDS, and N_2_ adsorption–desorption analyses differentiated the materials in terms of structure, morphology, elemental composition, and textural properties, the equal-mass design does not normalize particle volume, particle number, or the surface area accessible within the drilling fluid.

For each formulation, 929 mL of tap water was initially mixed with 0.3 g of sodium hydroxide for 1 min to adjust the alkalinity. Subsequently, 0.5 g of sodium carbonate was added and mixed for 1 min, followed by the addition of 70 g of potassium chloride, which was stirred for 2 min to ensure complete dissolution. Thereafter, 1 mL of defoamer and 1 mL of biocide were added sequentially, with each additive being mixed for 1 min under continuous agitation.

For WBM 1–WBM 3, the selected carbon-based material, grapevine-wood-derived carbon (GWC), graphitic carbon nitride (GCN), or commercial activated carbon (CAC), respectively—was then incorporated at an amount of 20 g per formulation and dispersed for 2 min. For the reference formulation (WBM 0), this incorporation step was omitted. Subsequently, 10 g of low-viscosity polyanionic cellulose (PAC-LV) was added and mixed for 5 min for fluid-loss control, followed by 3 g of xanthan gum, which was mixed for an additional 5 min to adjust the rheological properties. Finally, 203 g of barite was gradually introduced as a weighting agent, and mixing was continued for 42 min to achieve complete homogenization and the target drilling-fluid density.

All formulations were prepared at room temperature using a Hamilton Beach mixer (Hamilton Beach, Glen Allen, VA, USA) operated at speed setting 2. Following conventional mixing, the drilling fluids were subjected to high-shear homogenization using a Silverson mixer (Silverson, East Longmeadow, MA, USA) at 6000 rpm for 10 min to homogenize the formulations.

### 2.5. Characterization and Performance Testing of the Reference and Carbon-Modified Water-Based Drilling Fluids

The physicochemical, rheological, filtration, and shale-inhibition properties of the prepared water-based drilling fluids were systematically evaluated. To maintain methodological consistency with the drilling-fluid systems examined in our previous studies [20,21], the same analytical procedures, reagent concentrations, instruments, and operating conditions were employed throughout the present investigation. Maintaining a consistent experimental protocol minimized methodological variability and allowed differences in performance to be associated primarily with the absence or presence and type of carbon-based additive. WBM 0 served as the carbon-additive-free reference formulation, whereas WBM 1–WBM 3 contained grapevine-wood-derived carbon (GWC), graphitic carbon nitride (GCN), and commercial activated carbon (CAC), respectively. The alkalinity of the drilling fluids was determined according to API Recommended Practice 13B-1 by measuring the bicarbonate ($HCO_{3}^{-}$), carbonate ($CO_{3}^{2-}$), and hydroxide ($OH^{-}$) ion contents. Titrations were performed using 0.02 N sulfuric acid as the titrant, with phenolphthalein and bromocresol green serving as pH indicators.

The total hardness, expressed in terms of calcium and magnesium ion concentrations, was determined following the API RP 13B-1 procedure [22]. Complexometric titration was carried out using standardized disodium ethylenediaminetetraacetate (EDTA) solution as the titrant. A buffer solution containing ammonium chloride and ammonium hydroxide was employed to maintain the required pH, while calmagite (Calver II) was used as the colorimetric endpoint indicator [22].

Chloride concentration in the drilling-fluid filtrates was measured by argentometric titration. A 1 N silver nitrate solution was used as the titrant, and potassium chromate served as the endpoint indicator. Before titration, the pH of the filtrate was adjusted using 0.02 N sulfuric acid and phenolphthalein, in accordance with API RP 13B-1 [22]. All titrations were conducted on filtrate samples collected from the prepared drilling fluids at ambient temperature.

The density and pH of each formulation were measured to assess the physical state and acid–base characteristics of the fluids. Rheological measurements were conducted using a Fann Model 35 viscometer (Fann Instrument Company, Houston, TX, USA) after conditioning the drilling fluids at 49 °C. Dial readings were recorded at rotational speeds of 600, 300, 200, 100, 6, and 3 rpm. The 10 s and 10 min gel strengths were also determined to evaluate the time-dependent structure development and thixotropic recovery of the fluids. Plastic viscosity (PV), yield point (YP), and apparent viscosity (AV) were subsequently calculated from the viscometer readings to describe the flow behavior and suspension capacity of the formulations.

Fluid-loss behavior was evaluated using a conventional low-pressure, low-temperature (LPLT) API filter press with CO_2_ pressure assembly (OFITE, Houston, TX, USA) in accordance with ANSI/API Recommended Practice 13B-1, 4th edition (identical to ISO 10414-1:2008) [22]. The filter-press cell, with a filtration area of 45.8 cm^2^ (7.1 in^2^), was fitted with Whatman No. 50 filter paper supported by a screen. Immediately after preparation, without thermal aging or hot rolling, each formulation was poured into the cell, and a pressure of 100 psi (690 kPa) was applied using compressed CO_2_. The filtrate was collected for 30 min at ambient laboratory temperature, and its volume was reported in milliliters. After depressurization and disassembly of the cell, the filter cake was carefully recovered, gently washed to remove excess drilling fluid, and its thickness was measured in millimeters. Filter-cake permeability and post-filtration morphology were not determined.

The shale-inhibition performance of the four formulations was investigated by linear-swelling tests using a Dynamic Linear Swell Meter (OFITE, Houston, TX, USA) [23,24]. The shale wafers were prepared and tested using the same shale source, quantities, preparation procedure, equipment, and operating conditions employed in our previous investigations [20,21,25]. This approach enabled a consistent assessment of shale hydration and expansion in the carbon-additive-free and the effects produced by the incorporation of GWC, GCN, and CAC.

To further clarify the influence of the investigated carbon-based additives on the aqueous chemistry of the drilling fluids, the ionic composition of the collected filtrates was analyzed. Standard analytical procedures were used to determine the concentrations of $Ca^{2+}$, $Mg^{2+}$, $Cl^{-}$, $HCO_{3}^{-}$, $CO_{3}^{2-}$, and $OH^{-}$. Monitoring these ionic species was important because changes in ionic equilibria can affect clay hydration, particle dispersion, fluid stability, and overall shale-inhibition performance.

Comparisons among WBM 0–WBM 3 constitute direct within-study comparisons because these formulations were prepared and tested within the same experimental program using the same base-fluid composition, procedures, equipment, and operating conditions. Comparisons with the organic inhibitors and nanocrystalline metal oxides investigated in our previous studies [20,21] are presented only as contextual cross-study benchmarks. Although the additive dosage and most preparation and testing procedures were closely related, minor differences in base-fluid composition preclude interpreting these comparisons as direct head-to-head evaluations.

### 2.6. Linear Shale-Swelling Test

Following preparation, the four drilling-fluid formulations (WBM 0–WBM 3) were evaluated for shale-inhibition performance using a Dynamic Linear Swell Meter (OFITE, Houston, TX, USA) over 72 h. To facilitate contextual comparison with the results reported in our previous studies [20,21], the shale wafers were prepared and tested using exactly the same shale source, preparation procedure, equipment, and operating conditions.

The shale wafers were prepared from 15 g of shale cuttings (<500 μm) taken from the same stored batch used in our previous investigations [20,21]. This batch was collected from the X Rosetti Formation at a depth of 2917 m. Its mineralogical composition was previously determined by X-ray diffraction (XRD) and reported in our earlier studies [20,21]. The cuttings were compacted in a cell press at 6000 psi (approximately 41 MPa) for 30 min, producing wafers with a molded density of 2.6 g cm^−3^ and a diameter of 28 mm. Before testing, the wafers were conditioned for 24 h under controlled ambient conditions. One wafer was tested per formulation, except for WBM 2, for which three wafers from independently prepared batches were tested (Section 2.7). For the remaining formulations, the variability between wafers could not be determined, which is acknowledged as a limitation of the present study. Moreover, because the wafers were prepared from a single shale sample by compaction of cuttings, their pore structure, bedding, and fracture network differ from those of intact shale. The swelling results are therefore suitable for the relative comparison of the formulations but cannot be generalized to shales of different mineralogy, particularly different smectite contents.

Linear swelling measurements were conducted at 20–23 °C, and the percentage increase in wafer height was continuously monitored for 72 h. The swelling behavior observed in the carbon-additive-free reference formulation (WBM 0) was compared with that obtained for the formulations containing grapevine-wood-derived carbon (GWC), graphitic carbon nitride (GCN), and commercial activated carbon (CAC). The performances of the three carbon-modified formulations were also compared with one another to assess the relative shale-inhibition effectiveness of the investigated additives. Because the experimental conditions were maintained identical to those used in our previous studies [20,21], the previously reported results were used only as contextual cross-study benchmarks for the present findings.

### 2.7. Replication and Statistical Analysis

To evaluate experimental repeatability, the GCN-containing formulation (WBM 2) was prepared and tested in triplicate using three independently prepared batches (n = 3). Each batch was subjected to the complete preparation and testing sequence described in Section 2.4, Section 2.5 and Section 2.6, and a separate shale wafer was used for each linear-swelling test. The remaining formulations (WBM 0, WBM 1, and WBM 3) were prepared and tested once (n = 1). For WBM 2, PV, YP, and AV were calculated for each replicate from the corresponding Fann dial readings before averaging, and all results are reported as mean ± standard deviation (SD).

Because the remaining formulations were evaluated once, inferential statistical comparisons among the four formulations could not be performed. The SD obtained for WBM 2 was therefore used only as an indicative estimate of method repeatability, and differences between formulations smaller than approximately twice this SD were not interpreted as meaningful.

## 3. Results and Discussion

### 3.1. Properties of the Carbon-Based Materials

#### 3.1.1. X-Ray Diffraction Analysis

**Figure 1:**
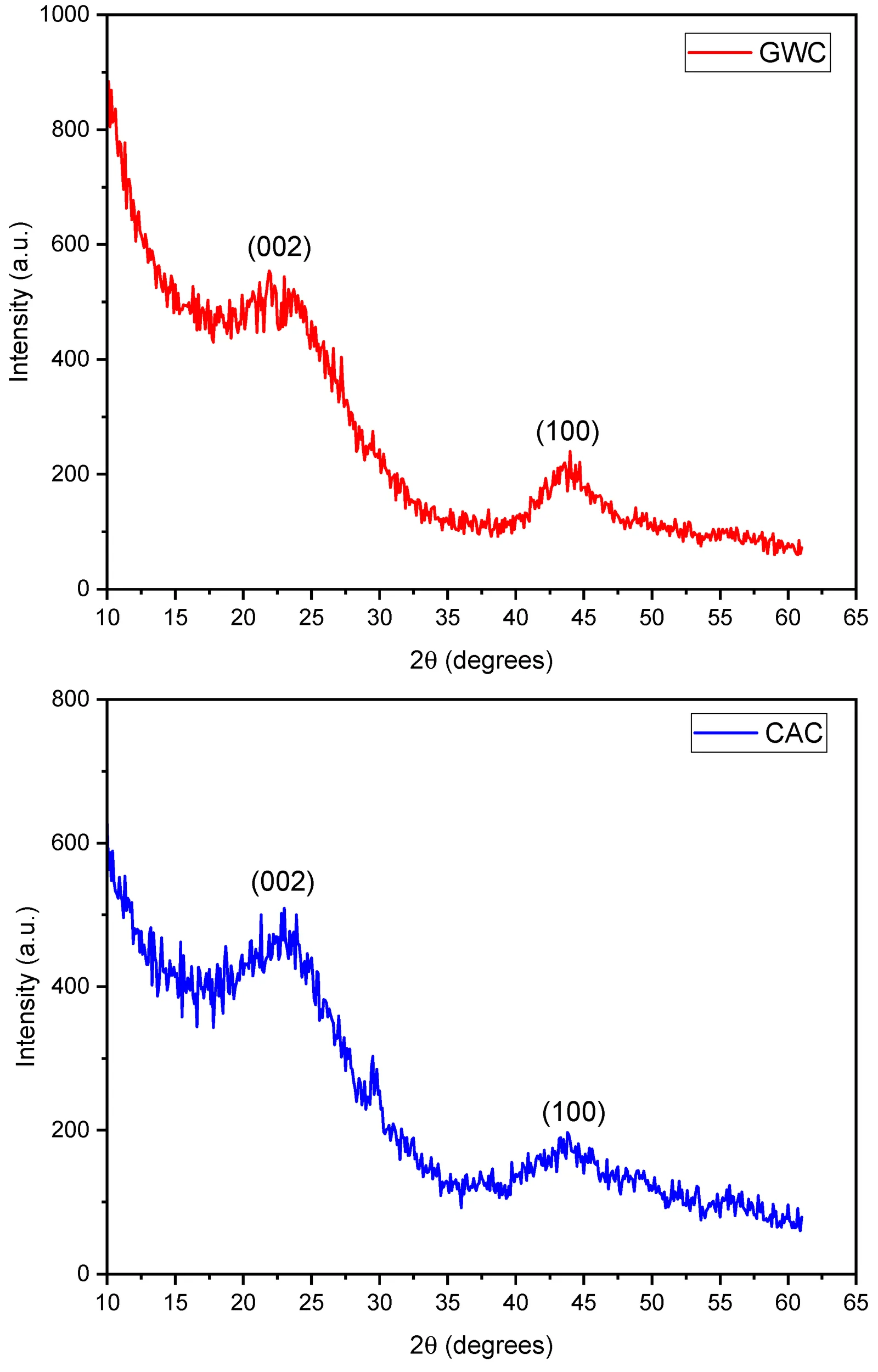

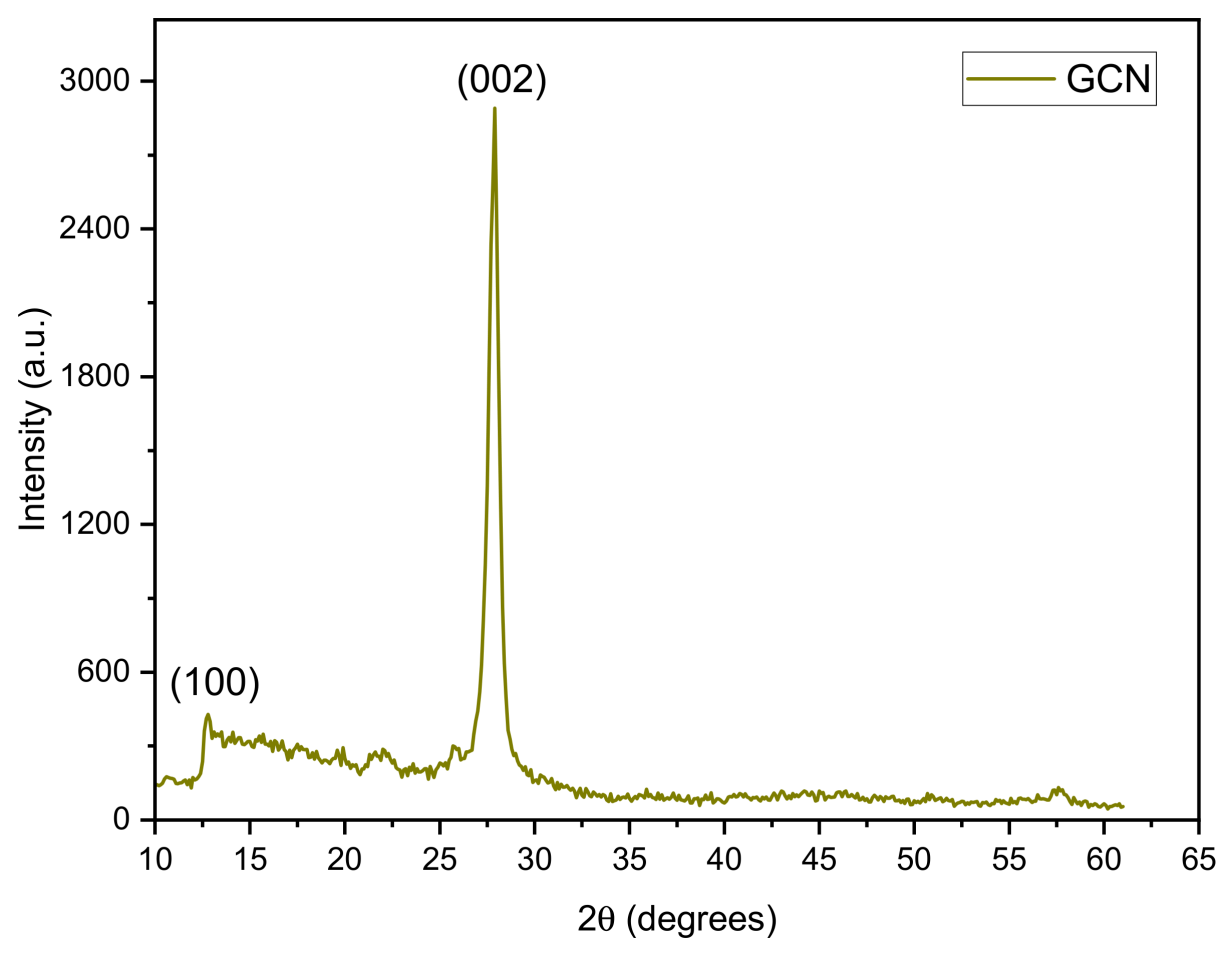
X-ray diffraction (XRD) patterns of GWC, CAC, and GCN.

The XRD patterns of GWC and CAC, as shown in Figure 1, exhibit two broad diffraction peaks centered at approximately 2θ~23.5° and 42.5°, corresponding to the (002) and (100) planes, respectively. These broad peaks are characteristic of predominantly disordered carbon structures composed of aromatic carbon layers with limited short-range ordering. Similar broad diffraction features within the ranges of 20–30° and 40–50° have been commonly reported for biochar and activated carbon materials [26,27,28,29].

In contrast, the GCN pattern shows two characteristic diffraction peaks at 2θ = 12.8° and 27.9°, assigned to the (100) and (002) planes of graphitic carbon nitride, respectively. The peak at 12.8° is associated with the in-plane structural arrangement of tri-s-triazine units, whereas the intense peak at 27.9° corresponds to the interlayer stacking of conjugated aromatic layers. The corresponding interplanar distances were 0.680 and 0.324 nm, respectively. These results confirm the formation of the characteristic layered structure of g-C_3_N_4_ synthesized by thermal condensation of melamine at 600 °C [30,31,32,33].

#### 3.1.2. N_2_ Adsorption–Desorption Analysis

**Figure 2:**
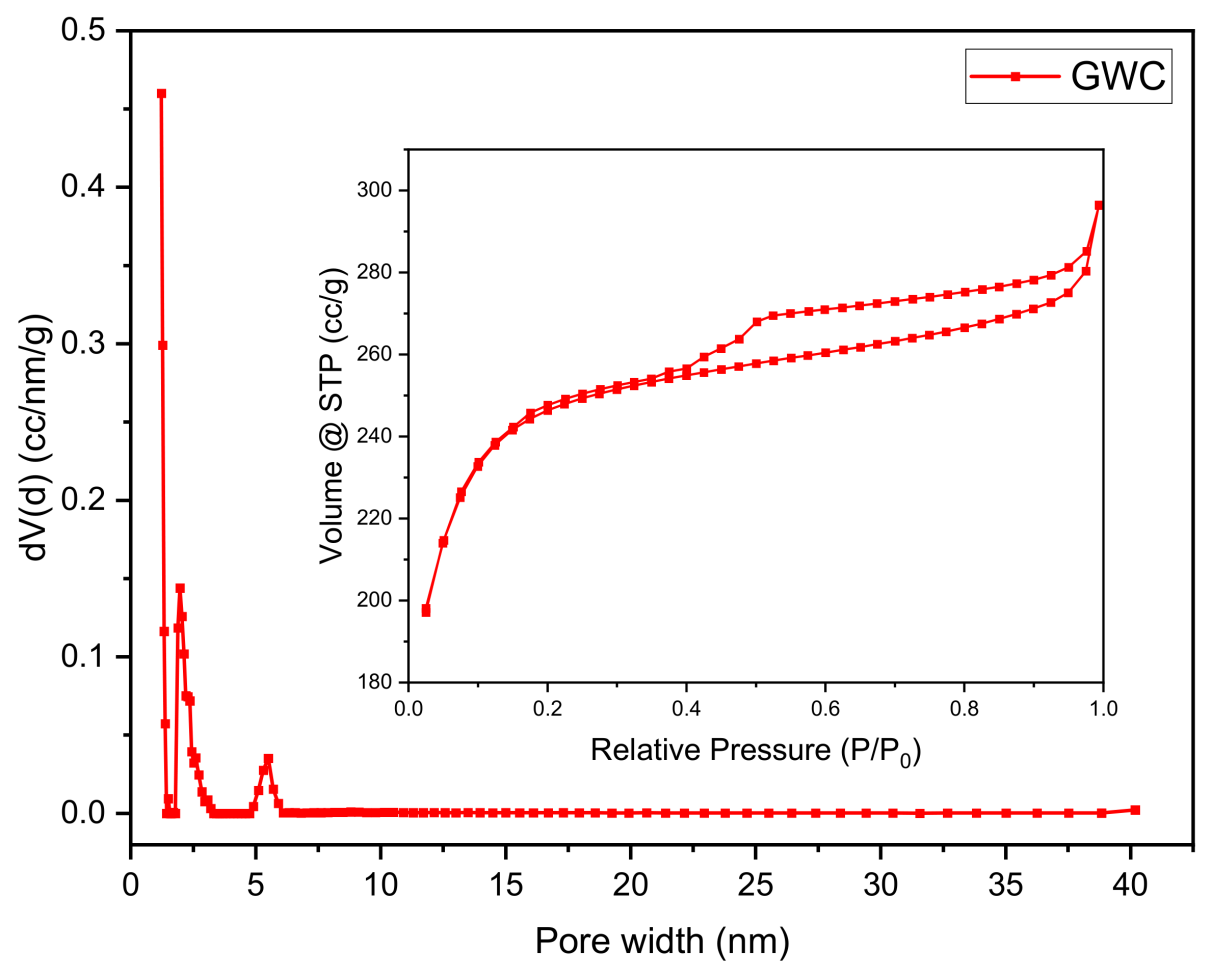

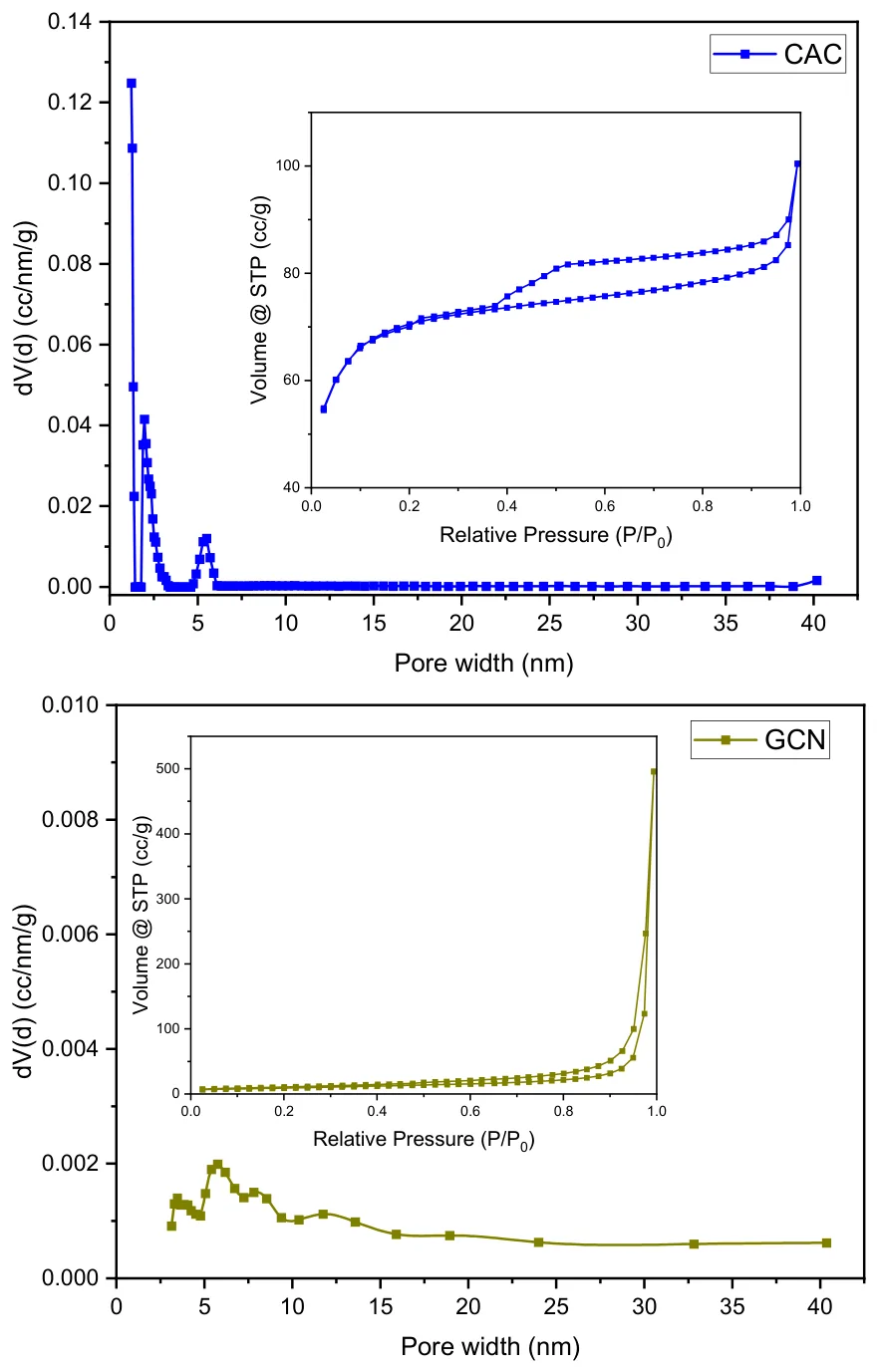
N2 adsorption–desorption isotherms and corresponding DFT pore size distributions of GWC, CAC, and GCN.

**Figure 3:**
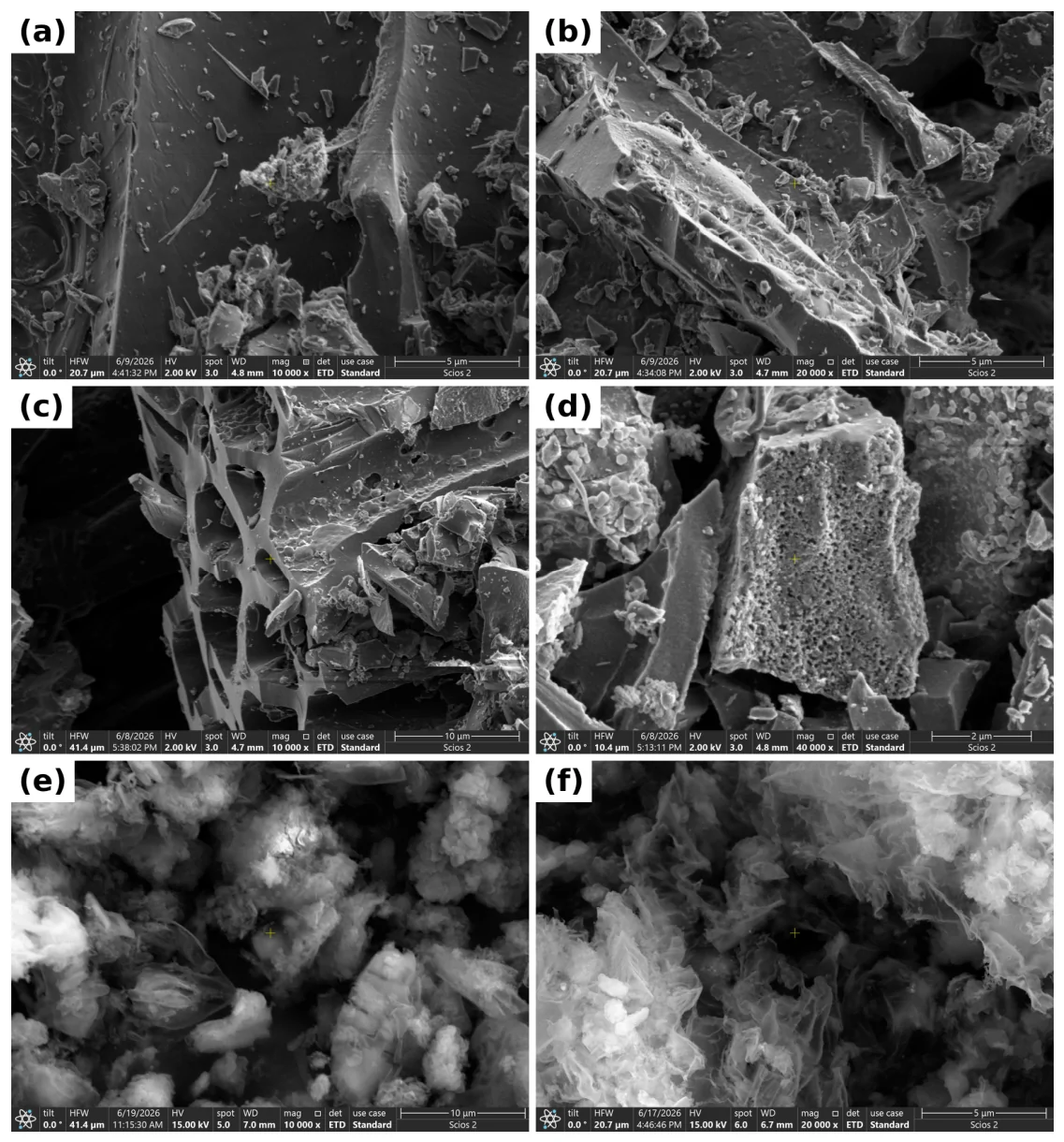
SEM micrographs of (**a**,**b**) GWC, (**c**,**d**) commercial activated carbon (CAC), and (**e**,**f**) GCN at different magnifications.

The N_2_ adsorption–desorption isotherms of GWC, CAC, and GCN are shown in Figure 2. GWC and CAC exhibit predominantly Type I isotherms with H4 hysteresis loops, indicating mainly microporous structures containing narrow slit-shaped pores [34,35,36,37]. In contrast, GCN displays a Type IV isotherm with an H3 hysteresis loop, commonly associated with mesopores and interparticle voids formed between aggregated plate-like particles [34,35,36,37]. This interpretation is consistent with the aggregated morphology observed in the SEM images (Figure 3) and the layered structure of GCN revealed by XRD analysis (Figure 1). The observed differences in the adsorption–desorption behavior therefore demonstrate the distinct pore architectures of the three materials, with microporosity prevailing in GWC and CAC and mesoporosity being more characteristic of GCN.

The textural properties obtained from the N_2_ adsorption–desorption analysis are summarized in Table 2. GWC exhibited the highest DFT-derived specific surface area and total pore volume, reaching 939.17 m^2^/g and 0.415 cm^3^/g, respectively. CAC showed lower values of 268.59 m^2^/g and 0.130 cm^3^/g, whereas GCN presented the lowest surface area (32.50 m^2^/g) and a total pore volume of 0.250 cm^3^/g. The pore-size distributions were determined using the density functional theory (DFT) model. The modal pore widths were 1.22 nm for both GWC and CAC, confirming the predominance of micropores, whereas GCN exhibited a modal pore width of 5.20 nm, corresponding to the mesopore range. Consistently, the mean slit-pore width estimated from the total pore volume and DFT surface area (w ≈ 2V/S) was approximately 0.9 nm for GWC (0.88 nm) and CAC (0.97 nm), but approximately 15 nm for GCN, indicating that the pore volume of GCN is associated with substantially wider pores. These findings are consistent with the Type I isotherms and pronounced N_2_ uptake observed at low relative pressures for GWC and CAC, as well as the Type IV isotherm obtained for GCN. Overall, GWC exhibited the most developed porous structure among the investigated materials, as evidenced by its substantially higher specific surface area and total pore volume.

#### 3.1.3. Morphological Characterization by SEM-EDS

The SEM micrographs of GWC, CAC, and GCN are presented in Figure 3. GWC (Figure 3a,b) exhibits a heterogeneous morphology composed of irregular carbonaceous fragments, elongated structures, and randomly distributed surface deposits. Relatively smooth regions coexist with cracks, cavities, and rough aggregates, suggesting the preservation of some structural features inherited from the carbon precursor. Similar heterogeneous morphologies, including irregular fragments and residual channel-like structures, have been reported for biomass-derived carbon materials [26,38,39]. Although the micropores cannot be directly resolved at the magnifications used, the observed cavities and surface irregularities complement the porous characteristics revealed by the N_2_ adsorption–desorption analysis.

CAC (Figure 3c,d), used as a commercial activated-carbon reference, displays irregular, angular, and fractured particles with rough surfaces containing numerous cavities, cracks, and open pore-like features. Comparable rough and heterogeneous morphologies containing cavities, hollows, and uneven surface regions have been reported for different commercial activated-carbon materials [40,41]. These morphological features are generally associated with the precursor characteristics and the carbonization, activation, and particle-processing conditions used during commercial production [40,42].

Although relatively large cavities are more clearly visible in the CAC micrographs, this does not indicate greater overall porosity than GWC, because the micropores that contribute substantially to the DFT-derived specific surface area cannot be resolved by SEM at the applied magnifications. Therefore, the predominantly microporous character of CAC is more reliably supported by the N_2_ adsorption–desorption results.

In contrast, GCN (Figure 3e,f) exhibits a distinctly different morphology consisting of densely aggregated, crumpled, and overlapping sheet-like structures. The stacking and random aggregation of these layers generate interparticle voids and a relatively loose, fluffy texture. Similar lamellar and aggregated sheet-like morphologies have been widely reported for bulk g-C_3_N_4_ synthesized through the thermal polycondensation of melamine [43,44]. This morphology is consistent with the layered structure indicated by the characteristic (002) diffraction peak in the XRD pattern. Furthermore, the interparticle voids formed between the aggregated sheets agree with the Type IV N_2_ adsorption–desorption isotherm and H3 hysteresis loop observed for GCN.

**Figure 4:**
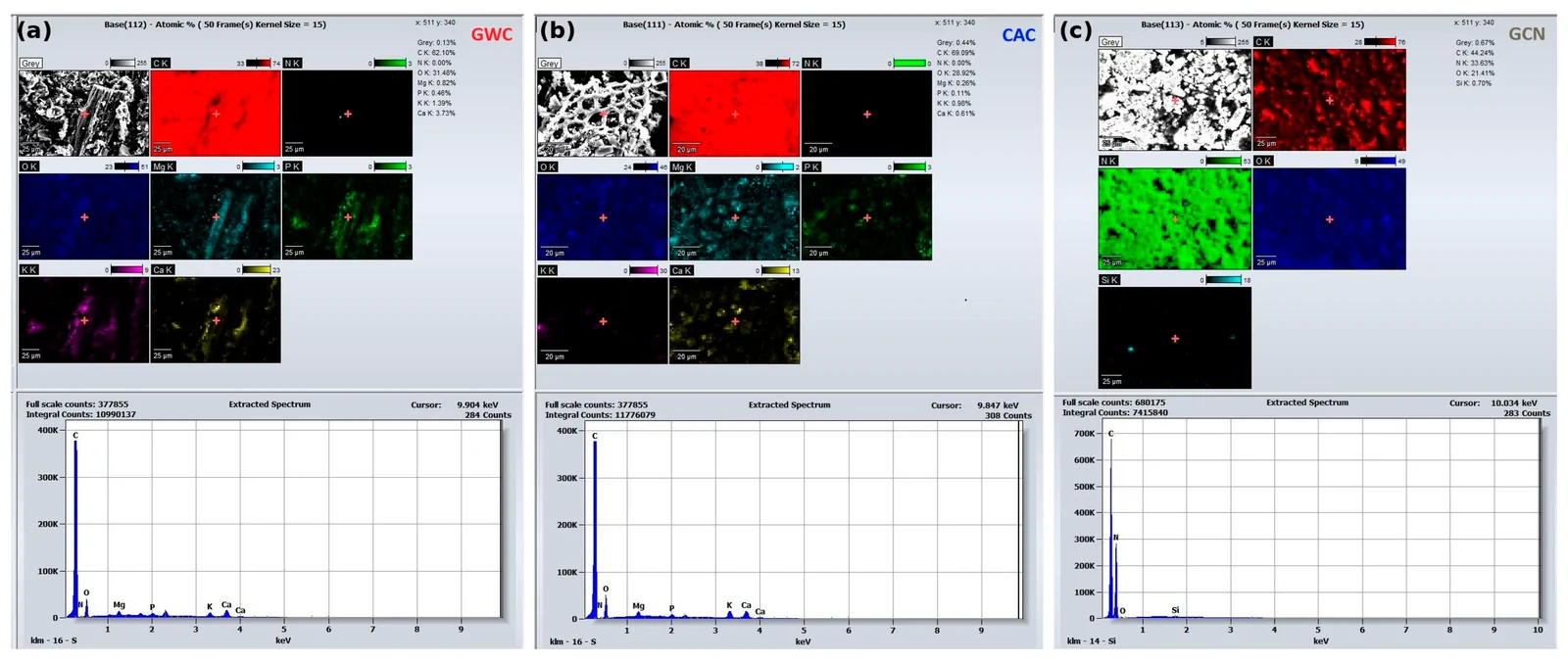
EDS elemental maps and spectra of (**a**) GWC; (**b**) commercial activated carbon (CAC); (**c**) GCN, showing the spatial distribution of the detected elements.

The elemental composition and spatial distribution of the detected elements in GWC, CAC, and GCN were investigated using EDS analysis and elemental mapping, as shown in Figure 4. The EDS results indicated that carbon was the predominant element in both GWC and CAC, consistent with their carbonaceous nature. In GWC, carbon was broadly distributed throughout the analyzed region, whereas oxygen and minor amounts of Mg, P, K, and Ca were also detected. These minor inorganic elements were heterogeneously distributed, with some localized enriched regions visible in the elemental maps. Their presence can reasonably be attributed to mineral constituents retained from the original biomass precursor following carbonization [45,46].

Similar SEM–EDS analyses of commercial activated carbons (CAC) have revealed carbon-rich compositions accompanied by minor inorganic constituents, including K and Ca [47]. Elements such as Ca, Mg, K, and P may originate from the mineral fraction of the carbon precursor and may be retained to varying degrees following carbonization and activation [45,47].

In contrast, GCN was composed primarily of carbon and nitrogen, consistent with the formation of a nitrogen-rich carbon nitride material. The co-distribution of these elements agrees with the composition expected for g-C_3_N_4_, whereas its characteristic layered structure was independently supported by the XRD results. Oxygen was also detected and may be associated mainly with surface hydroxyl groups formed through the reaction of structural defects generated under N_2_ with atmospheric oxygen upon subsequent exposure to air, in addition to adsorbed oxygen and water [48].

Overall, the EDS results clearly differentiate the carbon-dominated compositions of GWC and CAC from the nitrogen-rich C–N composition of GCN, while also revealing minor residual inorganic elements in the two carbonaceous materials.

The semi-quantitative EDS results obtained from one representative area of each material are summarized in Table 3. GWC and CAC consisted predominantly of carbon and oxygen, with minor contributions from Mg, P, K, and Ca, whereas GCN was composed mainly of carbon and nitrogen, together with oxygen and a minor amount of Si. Notably, Ca accounted for 3.73 at.% in the analyzed GWC area.

### 3.2. Mineralogical Composition of the X Rosetti Shale

**Figure 5:**
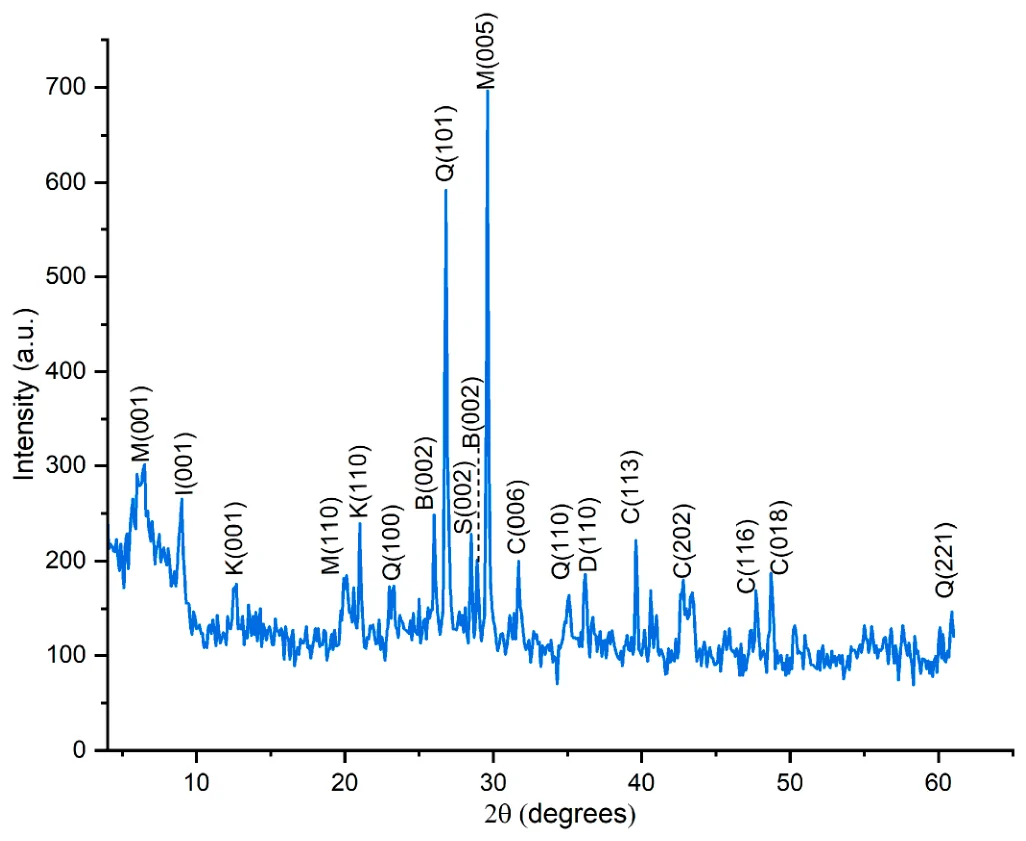
Previously reported XRD pattern of the X Rosetti shale, showing the principal crystalline mineral phases. Reproduced from Ref. [21].

For completeness and to provide the mineralogical context required for interpreting the linear-swelling behavior, the previously reported XRD pattern of the X Rosetti shale is reproduced in Figure 5. The mineralogical composition of this shale was previously determined by X-ray diffraction and reported in our earlier studies [20,21]. No additional XRD measurements were performed in the present study.

The diffractogram exhibits distinct crystalline reflections corresponding to several major and minor mineral phases. Characteristic reflections were assigned to quartz at 2θ = 23.5°, 26.8°, and 35.1°, corresponding to the (100), (101), and (110) planes, respectively [49,50]. Calcite reflections were identified at 31.2°, 39.3°, 43.1°, 47.4°, and 49°, corresponding to the (006), (113), (202), (116), and (018) planes [51,52]. Montmorillonite was identified from the reflections at 6.5°, 19.9°, and 29.6°, assigned to the (001), (110), and (005) planes, respectively [53]. Additional reflections were attributed to illite (001) at 9.6°; kaolinite (001) and (110) at 12.6° and 21°, respectively [54]; barite (002) and (210) at 26° and 28.5°, respectively [55]; and dolomite (110) at 36.2° [56]. The coexistence of these mineral phases confirms the heterogeneous and complex nature of the argillaceous matrix.

The previously reported quantitative phase analysis indicated that the shale was predominantly composed of calcite (approximately 30 wt.%) and montmorillonite (approximately 29 wt.%), followed by quartz (approximately 19 wt.%), illite (approximately 9 wt.%), kaolinite (approximately 4 wt.%), barite (approximately 4 wt.%), sylvite (approximately 4 wt.%), and dolomite (approximately 1 wt.%).

The relatively high montmorillonite content explains the susceptibility of the X Rosetti shale to water uptake and swelling upon exposure to water-based drilling fluids. Montmorillonite is an expandable clay mineral capable of hydrating and increasing its interlayer spacing, whereas illite and kaolinite are comparatively non-expanding clay phases. Conversely, the considerable proportions of calcite and quartz contribute to the mineralogical rigidity of the shale matrix but do not eliminate the swelling tendency associated with the expandable clay fraction. Because the shale wafers employed in the present and previous investigations were prepared from the same stored batch of shale cuttings collected from the same formation and sampling depth, the previously reported mineralogical data were considered representative of the samples tested herein. This mineralogical composition therefore provides an appropriate basis for evaluating and comparing the shale-inhibition performance of the GWC-, GCN-, and CAC-containing drilling fluids.

### 3.3. Performance Evaluation of Carbon-Modified Water-Based Drilling Fluids

The physicochemical, rheological, filtration, ionic, and shale-inhibition properties of the reference and carbon-modified water-based drilling fluids are summarized in Table 4; the results for WBM 2 are reported as mean ± SD (n = 3). WBM 0, which contained no additional carbon-based material, was used as the reference formulation, whereas WBM 1, WBM 2, and WBM 3 contained GWC, GCN, and CAC, respectively. All formulations exhibited the same mud weight of 1.20 g/cm^3^, indicating that the incorporation of 20 g of carbon-based material did not measurably affect the final drilling-fluid density. This value was also identical to that reported for the water-based drilling fluids containing the imidazoline derivatives and commercial inhibitors in our previous study [21] and the nanocrystalline metal oxides investigated in our subsequent work [20]. Consequently, differences in the measured performance parameters cannot be attributed to variations in fluid density.

The pH values remained within a relatively narrow alkaline range of 9.2–9.4. WBM 2 containing GCN exhibited a mean pH of 9.35 ± 0.09 (n = 3), which differed from that of the reference formulation by less than twice the SD, while the reference and GWC-containing formulations showed a pH of 9.3, whereas WBM 3 containing CAC showed a slightly lower value of 9.2. The limited variation indicates that the investigated carbon-based additives did not substantially disturb the alkalinity established by sodium hydroxide and sodium carbonate. The pH values were comparable to those previously obtained for WBMs containing Amine NF, Glycol, A-Lau, and A-Lin (9.20–10.23) [21] and nanocrystalline Fe_2_O_3_, CuO, ZnO, and MgO (9.0–10.0) [20]. Maintaining a mildly alkaline environment is advantageous for drilling-fluid stability and may also limit excessive clay dispersion.

The triplicate measurements of WBM 2 showed good repeatability for most parameters, with coefficients of variation of 2.3% for API filtrate volume, 2.8% for final linear swelling, and 2.1% for AV. Larger relative variability was observed for PV (11.4%) and particularly for YP (23.1%). Because these parameters are calculated from differences between dial readings, small variations in the individual readings are amplified in the calculated values. Relative to these SDs, the differences between WBM 2 and WBM 0 in final linear swelling (4.9 percentage points), API filtrate volume (0.9 mL), mud-cake thickness (0.55 mm), PV (6.3 mPa·s), and AV (3.8 mPa·s) exceeded twice the corresponding SD, whereas the differences in YP (5.0 lb/100 ft^2^) and pH (0.05) did not.

Accordingly, the YP values of the four formulations (15.0–22 lb/100 ft^2^) are regarded as comparable within the observed variability (SD = 3.5 lb/100 ft^2^ for WBM 2), and the higher YP of WBM 3 is not interpreted as a meaningful difference. Similarly, the differences in PV among WBM 0, WBM 1, and WBM 3 (13–17 mPa·s) do not exceed approximately twice the SD obtained for WBM 2 and are therefore not considered conclusive. In contrast, the reduction in API filtrate volume obtained with GWC (2.4 mL relative to WBM 0) and the reductions in final swelling obtained with all three carbon-based additives (3.0–4.9 percentage points) substantially exceed the corresponding SDs (0.2 mL and 0.2%, respectively).

The rheological measurements revealed distinct effects of the three carbon-based materials. Relative to WBM 0, GWC slightly decreased the high-shear Fann readings from 48 to 44 at 600 rpm and from 34 to 31 at 300 rpm. Accordingly, the plastic viscosity decreased from 14 to 13 mPa·s, while the apparent viscosity decreased from 24.0 to 22.0 mPa·s. These changes may reflect reduced resistance to flow and less extensive particle–particle or particle–polymer structuring under the tested conditions. By contrast, GCN increased the 600 and 300 rpm readings to 55.7 ± 1.2 and 35.3 ± 1.2, respectively, producing a plastic viscosity of 20.3 ± 2.3 mPa·s and an apparent viscosity of 27.8 ± 0.6 mPa·s. CAC produced the highest 600 rpm reading (56) and apparent viscosity (28.0 mPa·s), together with a plastic viscosity of 17 mPa·s. The higher PV and AV values of the GCN- and CAC-containing formulations indicate greater resistance to flow under the tested conditions. Particle–particle and particle–polymer interactions may contribute to these changes, but their individual contributions cannot be identified from the rheological data alone.

For contextual reference, the plastic viscosities of the present carbon-modified formulations (13–20.3 mPa·s) fell within or close to the ranges previously reported for formulations containing Amine NF, Glycol, A-Lau, and A-Lin (14–22 mPa·s) [21] and Fe_2_O_3_, CuO, ZnO, and MgO (17–19 mPa·s) [20].

The yield-point values were 20, 18, 15.0 ± 3.5, and 22 lb/100 ft^2^ for WBM 0, WBM 1, WBM 2, and WBM 3, respectively. However, these differences did not exceed approximately twice the SD obtained for the replicated WBM 2 formulation (SD = 3.5 lb/100 ft^2^); therefore, the yield points of the four formulations are regarded as comparable within the observed variability. The yield-point values of all four formulations also fell within the range previously reported for the inhibitor- and oxide-containing systems [18,19]. The 10 s/10 min gel strengths were 6/8, 5/6, 6/7, and 6/8 lb/100 ft^2^ for WBM 0, WBM 1, WBM 2, and WBM 3, respectively. The moderate increase between the initial and 10 min values indicates controlled thixotropic recovery without excessive gel development. GWC produced the weakest gel structure, consistent with its lower PV, AV, and high-shear Fann readings.

Marked differences were observed in filtration behavior. The reference formulation produced an API filtrate volume of 8.0 mL and a mud-cake thickness of 1.5 mm. Incorporation of GWC reduced the filtrate volume to 5.6 mL, corresponding to a 30.0% reduction, while decreasing the mud-cake thickness to 0.5 mm.

The simultaneous reduction in filtrate volume and mud-cake thickness demonstrates improved filtration performance under the tested conditions. This behavior may be associated with more effective particle packing and sealing within the filtration layer. The irregular morphology, high DFT-derived specific surface area (939.17 m^2^/g), high total pore volume (0.415 cm^3^/g), and predominantly microporous structure of dry GWC may have contributed to this response. However, the structure and permeability of the resulting filter cake were not directly characterized; therefore, the respective contributions of these material properties to filtration control cannot be established from the present measurements.

For contextual reference, the API filtrate volume of the GWC-containing fluid (5.6 mL) was numerically identical to that previously reported for the Amine NF formulation and lower than the values reported for Glycol (6.0 mL), A-Lau (6.4 mL), and A-Lin (8.4 mL) [21]. It was also numerically lower than the values reported for Fe_2_O_3_ (6.0 mL), CuO (8.4 mL), ZnO (9.0 mL), and MgO (9.0 mL) [20], and its 0.5 mm mud cake was equal in thickness to those reported for Amine NF, Glycol, A-Lau, and all four oxide-containing systems [20,21]. Because the base-fluid formulations were not fully identical across studies, these values are cited as benchmarks rather than as evidence of superior performance. In contrast, GCN increased the filtrate volume to 8.9 ± 0.2 mL and produced the thickest mud cake (2.05 ± 0.13 mm).

The combination of relatively high fluid loss and increased mud-cake thickness indicates less effective filtration control than that obtained with GWC. The loose, layered morphology of dry GCN and the interparticle voids suggested by its Type IV isotherm and H3 hysteresis loop may have contributed to the observed behavior. However, because the morphology and permeability of the GCN-containing filter cake were not directly examined, the formation of an internally open filtration layer remains a possible interpretation rather than a demonstrated mechanism. For contextual reference, the GCN filtrate volume (8.9 ± 0.2 mL) lay between the values previously reported for A-Lin and CuO (8.4 mL) and those reported for ZnO and MgO (9.0 mL) [20,21], whereas its 2.05 ± 0.13 mm mud cake was numerically thicker than those reported for all previously evaluated systems.

CAC exhibited intermediate filtration performance. Its API filtrate volume decreased to 7.5 mL, representing a 6.25% reduction relative to WBM 0, while mud-cake thickness decreased from 1.5 to 1.0 mm. Because this 0.5 mL reduction only marginally exceeds twice the SD obtained for the replicated WBM 2 formulation (0.2 mL), it should be interpreted with caution.

Although CAC and GWC both exhibited predominantly microporous structures with a modal pore width of 1.22 nm, CAC possessed a substantially lower specific surface area and total pore volume. CAC produced a smaller improvement in filtration control than GWC under the tested conditions. However, because the morphology and permeability of the resulting filter cakes were not directly characterized, this difference cannot be attributed conclusively to the specific surface area, pore volume, or morphology of the dry additives.

Although the dry-material morphology observed by SEM may influence particle packing and interactions within the drilling fluid, the dispersion and aggregation states of the additives after mixing were not measured directly. Therefore, their contributions to the observed rheological and filtration behavior remain tentative.

The ionic analyses showed that all formulations maintained the same chloride concentration of 33,000 mg/L (33,033 ± 58 mg/L for WBM 2), consistent with their identical KCl content, while hydroxide ions were not detected. In contrast, substantial differences were observed in the titration-derived bicarbonate and carbonate concentrations. Bicarbonate increased from 610 mg/L in WBM 0 to 976, 1210 ± 26, and 1708 mg/L in the GWC-, GCN-, and CAC-containing formulations, respectively, whereas carbonate decreased from 480 mg/L to 96, 360 ± 15, and 120 mg/L. Because all four formulations contained identical initial amounts of sodium hydroxide and sodium carbonate, these differences coincided with the presence and type of carbon-based additive. Their origin, however, cannot be assigned conclusively. Possible contributions include pH-dependent redistribution between carbonate species [57], atmospheric CO_2_ uptake during preparation or handling, acid–base interactions with the additive surfaces, additive–polymer interactions, and the release or exchange of inorganic constituents. Aqueous release of inorganic carbon, alkalinity, and organic acids from biochar has previously been shown to depend strongly on the biomass feedstock and pyrolysis conditions [58].

Calcium concentration increased from 40 mg/L in WBM 0 to 96, 83 ± 3, and 100 mg/L in the GWC-, GCN-, and CAC-containing formulations, respectively. Magnesium remained at 48 mg/L in WBM 0, GWC, and CAC but increased to 72 ± 3 mg/L in the GCN-containing fluid. For GWC, the detection of 3.73 at.% Ca by EDS, together with the increased calcium concentration in the filtrate, is consistent with the possible release or exchange of calcium-containing species. Nevertheless, EDS establishes only the presence of calcium in the solid material and does not determine its chemical form, solubility, or transfer to the aqueous phase. Moreover, the similarly elevated calcium concentration and the highest bicarbonate concentration observed with CAC indicate that the ionic changes cannot be attributed solely to the residual inorganic constituents detected in GWC.

Because the carbonate and bicarbonate concentrations were calculated from acid–base titration endpoints rather than determined by direct species-specific instrumental analysis, contributions from other acid-consuming constituents cannot be excluded. Additive-only leaching tests, total inorganic carbon measurements, and direct aqueous-phase analyses were not performed. Accordingly, the observed ionic differences are interpreted as formulation-level effects and are not considered direct evidence of mineral dissolution or of a specific shale-inhibition mechanism.

**Figure 6:**
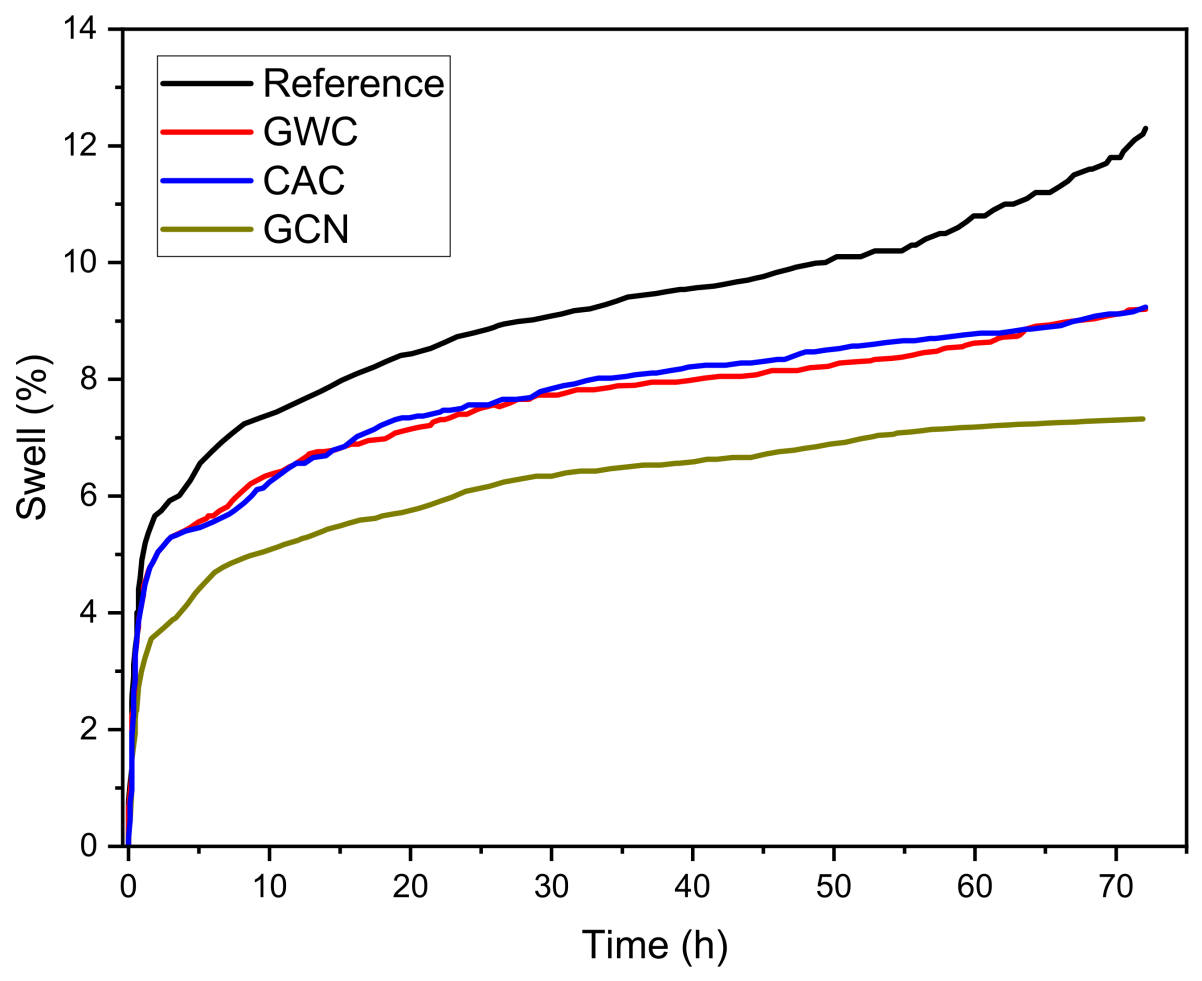
Time-dependent swelling profiles of X Rosetti shale exposed to the reference and carbon-modified water-based drilling fluids over 72 h.

The time-dependent shale-swelling profiles of the reference and carbon-modified drilling fluids over the 72 h test period are presented in Figure 6. The most important differences were observed in the linear shale-swelling tests. At the end of the 72 h test, the reference formulation produced 12.2% swelling. Incorporation of GWC and CAC reduced the final swelling to 9.2%, corresponding to a reduction of approximately 24.6% relative to the carbon-additive-free reference formulation (WBM 0). Among the tested additives, GCN produced the greatest reduction in final shale swelling, decreasing it to 7.3 ± 0.2% (n = 3), equivalent to a 39.9% reduction relative to WBM 0. Therefore, the inhibition ranking was:

(1) GCN>GWC≈CAC>WBM 0

The lower final swelling obtained with GCN is particularly notable because this material possessed the lowest DFT-derived specific surface area (32.50 m^2^/g) among the investigated materials. This result demonstrates that shale inhibition was not governed solely by specific surface area or micropore volume. The nitrogen-rich composition, layered structure, and aggregated plate-like morphology of GCN could favor interfacial interactions or particle deposition at the shale surface. Previous studies have reported close interfacial contact and electrostatic interactions between g-C_3_N_4_ nanosheets and montmorillonite layers [17,59]. Moreover, the incorporation of g-C_3_N_4_ into water-based drilling fluids was previously associated with decreased accessible surface area and pore volume of the treated shale, consistent with surface coverage and partial pore blocking in that system [17]. However, no post-exposure XRD analysis, surface imaging, zeta-potential measurement, or particle-adsorption measurement was performed in the present study. Therefore, the partial obstruction of water-penetration pathways and the restriction of water access to expandable clay interlayers are proposed only as plausible interpretations of the measured reduction in swelling.

GWC and CAC reduced swelling to the same final value despite their substantially different specific surface areas and pore volumes. Their lower inhibition efficiency relative to GCN suggests that microporosity and high surface area alone were insufficient to maximize shale stabilization. GWC provided excellent filtration control but only moderate swelling inhibition, whereas GCN produced the strongest swelling inhibition despite relatively high fluid loss and a thick mud cake. This contrasting behavior suggests that bulk filtration control and clay-hydration inhibition may depend on partially different material properties. Under the investigated conditions, GWC was more effective in controlling filtration, whereas GCN produced the greatest reduction in shale swelling. The microscopic mechanisms responsible for these differences cannot be established directly from the present measurements.

For contextual reference, the final swelling obtained with GCN (7.3 ± 0.2%) was numerically lower than the value previously reported for Glycol (7.7%) but higher than those reported for A-Lau (7.0%), Amine NF (6.7%), and A-Lin (6.3%) [21], whereas the value of 9.2% obtained with GWC and CAC was numerically higher than all four. Although all additives in these studies were incorporated at 20 g per formulation and tested using closely related procedures, differences in additive chemistry and base-fluid composition preclude a direct ranking of the carbon-based materials against these inhibitors. These cross-study observations provide contextual benchmarks only and do not constitute direct performance comparisons.

Similarly, the final swelling values obtained in the present study (7.3–9.2%) were numerically higher than the range of 6.1–7.0% previously reported for the nanocrystalline metal oxides at the same additive amount [20]. In that study, the lowest swelling was reported for MgO (6.1%) and was attributed to the combined effects of physical pore plugging and ionic double-layer compression associated with released magnesium-containing species. Unlike MgO, the investigated carbon-based materials were not associated with the same pronounced increase in dissolved magnesium concentration. Their inhibition behavior may instead be related to particle deposition, surface interactions, morphology, aggregation state, or the physical restriction of water ingress; however, these possible mechanisms were not directly verified in the present study. More generally, the results represent a formulation-level comparison at the selected mass dosage and should not be interpreted as establishing intrinsic material superiority or an optimum dosage. Particle-size distribution, bulk density, surface functional groups, ash content zeta potential, wettability, and the dispersion and sedimentation behavior of the additives in the complete fluid were not determined, limiting further normalization of the comparison. In addition, all tests were performed without thermal aging (hot rolling), HTHP filtration, or high-temperature/high-pressure rheology, and properties relevant to field performance, such as lubricity, barite sag, cuttings-transport efficiency, contamination tolerance, and long-term stability, were not evaluated. Because downhole temperature, pressure, and circulation may alter additive dispersion, polymer stability, and filter-cake structure, the present results should not be extrapolated to field conditions, and PV and YP are used here only as laboratory rheological indicators. Overall, the three carbon-based materials produced different responses. Under the investigated conditions, GWC provided the best filtration control and the lowest apparent viscosity, GCN produced the greatest reduction in shale swelling, and CAC exhibited intermediate filtration behavior, while the yield points of all formulations were comparable within the observed variability.

## 4. Conclusions

The three carbon-based materials produced distinct responses when evaluated at the same dosage and under identical formulation and testing conditions. GWC provided the best filtration performance, reducing API fluid loss from 8.0 to 5.6 mL and mud-cake thickness from 1.5 to 0.5 mm, while producing the lowest apparent viscosity (22.0 mPa·s).

For contextual reference, the fluid-loss value obtained with GWC was numerically identical to that previously reported for Amine NF and slightly lower than that reported for Fe_2_O_3_. GCN showed less favorable filtration behavior but produced the lowest final shale swelling, decreasing it from 12.2% to 7.3 ± 0.2% (n = 3), corresponding to a 39.9% reduction relative to WBM 0. Also for contextual reference, this final swelling value was numerically lower than the previously reported value for Glycol but higher than those reported for the other organic inhibitors and nanocrystalline oxides. These cross-study comparisons are provided only as contextual benchmarks because the base-fluid formulations were not fully identical. GWC and CAC both reduced swelling to 9.2%, while CAC produced intermediate filtration performance and a yield point comparable to those of the other formulations within the observed variability.

These results suggest that bulk filtration control and shale-hydration inhibition may depend on partly different material properties; however, the microscopic mechanisms underlying the observed differences were not directly investigated. Under the investigated laboratory conditions, GWC provided the strongest fluid-loss control, whereas GCN produced the largest reduction in shale swelling. These findings are limited to the selected additive dosage, base-fluid formulation, shale sample, and laboratory testing conditions, without thermal aging or HTHP testing. Using grapevine residues as a carbon precursor offers a way to reuse agricultural waste. However, this study did not assess the carbon yield, energy, nitrogen and solvent demand, or cost of the synthesis routes, nor environmental performance, toxicity, leaching, biodegradability, or life-cycle impacts. Therefore, the results do not show that GWC is environmentally superior to conventional drilling-fluid additives.

## Figures and Tables

**Table 1:** Composition of the reference and carbon-based-material-modified water-based drilling fluids.

Product	WBM 0		WBM 1		WBM 2		WBM 3	
	Amount	wt.%	Amount	wt.%	Amount	wt.%	Amount	wt.%
Tap water (mL)	929	76.285	929	75.053	929	75.053	929	75.053
Caustic soda (g)	0.3	0.025	0.3	0.024	0.3	0.024	0.3	0.024
Sodium carbonate (g)	0.5	0.041	0.5	0.040	0.5	0.040	0.5	0.040
Potassium chloride (g)	70	5.748	70	5.655	70	5.655	70	5.655
Defoamer (mL)	1	0.082	1	0.081	1	0.081	1	0.081
Bactericide (mL)	1	0.082	1	0.081	1	0.081	1	0.081
GWC	–	–	20	1.616	–		–	–
GCN	–	–	–	–	20	1.616	–	–
CAC	–	–	–	–	–	–	20	1.616
PAC-LV (g)	10	0.821	10	0.808	10	0.808	10	0.808
Xanthan gum (g)	3	0.246	3	0.242	3	0.242	3	0.242
Barite (g)	203	16.669	203	16.400	203	16.400	203	16.400
Total	1217.8	100	1237.8	100	1237.8	100	1237.8	100

Note: GWC: grapevine-wood-derived carbon; GCN: graphitic carbon nitride; CAC: commercial activated carbon; PAC-LV: low-viscosity polyanionic cellulose. Weight percentages were calculated relative to the total mass of each formulation (1217.8 g for WBM 0 and 1237.8 g for WBM 1–WBM 3), assuming a density of approximately 1 g mL^−1^ for the liquid additives. Minor differences may arise from rounding.

**Table 2:** DFT-derived specific surface area, total pore volume, and modal pore width of GWC, CAC, and GCN.

Sample	Total Pore Volume (cm^3^/g)	DFT Specific Surface Are (m^2^/g)	Modal Pore Width (nm)
GWC	0.415	939.17	1.22
GCN	0.250	32.50	5.20
CAC	0.130	268.59	1.22

**Table 3:** Semi-quantitative elemental composition determined by EDS over one representative mapped area of each carbon-based material.

Element (at.%)	GWC	CAC	GCN
C	62.10	69.09	44.24
N	0.00	0.00	33.63
O	31.48	28.92	21.41
Mg	0.82	0.26	—
P	0.46	0.11	—
K	1.39	0.98	—
Ca	3.73	0.61	—
Si	—	—	0.70

Note: Values are expressed as atomic percentages (at.%); “—” indicates that the element was not identified.

**Table 4:** Properties and performance of the reference and carbon-modified water-based drilling fluids. WBM 2 values are reported as mean ± SD of three independently prepared batches (n = 3); all other values are single determinations (n = 1).

Parameters	WBM 0 Reference	WBM 1 GWC	WBM 2 GCN ᵃ	WBM 3 CAC
Mud weight (g/cm^3^)	1.20	1.20	1.20 ± 0.01	1.2
pH	9.3	9.3	9.35 ± 0.09	9.2
Fann 600 rpm @ 49 °C	48	44	55.7 ± 1.2	56
Fann 300 rpm @ 49 °C	34	31	35.3 ± 1.2	39
Fann 200 rpm @ 49 °C	27	25	30.7 ± 1.2	31
Fann 100 rpm @ 49 °C	19	17	19.7 ± 0.6	23
Fann 6 rpm @ 49 °C	6	8	6.3 ± 0.6	6
Fann 3 rpm @ 49 °C	5	6	5.3 ± 0.6	5
Gels 10″/10′	6/8	5/6	6/7	6/8
Plastic Viscosity (mPa·s)	14	13	20.3 ± 2.3	17
Yield Point (lb/100 ft^2^)	20	18	15.0 ± 3.5	22
API Filtrate (mL)	8	5.6	8.9 ± 0.2	7.5
Mud Cake (mm)	1.5	0.5	2.05 ± 0.13	1
Bicarbonate (mg/L)	610	976	1210 ± 26	1708
Carbonate (mg/L)	480	96	360 ± 15	120
Hydroxyl (mg/L)	0	0	0	0
Calcium (mg/L)	40	96	83 ± 3	100
Magnesium (mg/L)	48	48	72 ± 3	48
Chlorides (mg/L)	33,000	33,000	33,033 ± 58	33,000
AV (mPa·s)	24	22	27.8 ± 0.6	28
Swell %	12.2	9.2	7.3 ± 0.2	9.2

^a^ Mean ± standard deviation (n = 3). PV, YP, and AV were calculated for each replicate from the corresponding dial readings (PV = θ_600_ − θ_300_; YP = θ_300_ − PV; AV = θ_600_/2) and then averaged.

## Data Availability

The original contributions presented in this study are included in the article. Further inquiries can be directed to the corresponding authors.
